# Circulating Antimicrobial Peptides as Biomarkers of Inflammation and Airway Dysfunction After Marathon Running

**DOI:** 10.3390/biology14070825

**Published:** 2025-07-07

**Authors:** Marie-Therese Lingitz, Hannes Kühtreiber, Lisa Auer, Michael Mildner, Claus G. Krenn, Clemens Aigner, Bernhard Moser, Christine Bekos, Hendrik Jan Ankersmit

**Affiliations:** 1 Division of General Anesthesia and Intensive Care Medicine, Department of Anesthesia, Critical Care and Pain Medicine, Medical University of Vienna, 1090 Vienna, Austria; marie-therese.lingitz@meduniwien.ac.at (M.-T.L.); claus.krenn@meduniwien.ac.at (C.G.K.); 2 Applied Immunology Laboratory, Department of Thoracic Surgery, Medical University of Vienna, 1090 Vienna, Austrialisa.auer@meduniwien.ac.at (L.A.); clemens.aigner@meduniwien.ac.at (C.A.); bernhard.moser@meduniwien.ac.at (B.M.); 3 Comprehensive Center for Chest Diseases, Medical University of Vienna, 1090 Vienna, Austria; 4 Aposcience AG, 1200 Vienna, Austria; 5 Department of Dermatology, Medical University of Vienna, 1090 Vienna, Austria; michael.mildner@meduniwien.ac.at; 6 Department of Obstetrics and Gynecology, Medical University of Vienna, 1090 Vienna, Austria; christine.bekos@meduniwien.ac.at

**Keywords:** antimicrobial peptides, airway inflammation, exercise-induced bronchoconstriction

## Abstract

Marathon running exerts physical stress and may lead to transient immune dysregulation, increasing susceptibility to airway inflammation and exercise-induced bronchoconstriction (EIB). This study investigated systemic levels of antimicrobial peptides in athletes and their association with EIB. Serum concentrations of angiogenin, human beta-defensin 2 (hBD-2), major basic protein (MBP), S100A8, and S100A8/A9 were measured in 34 marathoners and 36 half-marathoners at baseline, immediately after a race, and seven days postrace using enzyme-linked immunosorbent assays and compared with 30 sedentary controls. Lung function was assessed by spirometry to identify bronchoconstriction. Levels of hBD-2 and S100A8/A9 were significantly elevated postrace in runners compared to baseline and controls, returning to baseline during recovery. During recovery, S100A8 levels remained slightly elevated in marathoners with EIB. Similarly, human beta-defensin 2 was modestly increased in runners who developed bronchoconstriction. Notably, S100A8 levels correlated negatively with lung function parameters, including forced expiratory volume and mid-expiratory flows. These findings suggest that endurance running induces systemic inflammatory responses and modulates innate immune peptides, particularly in individuals prone to bronchoconstriction. These peptides may serve as biomarkers of respiratory stress and help guide personalized strategies in endurance sports.

## Simple Summary

Endurance sports such as marathon running are becoming increasingly popular, but they exert intense strain on the body. This physical stress can temporarily weaken the immune system and affect the lungs, occasionally causing breathing problems after exercise. Our study investigated whether specific substances circulating in the blood—antimicrobial peptides, which help the body fight off infections—change after endurance exercise and if they are linked to breathing issues occurring thereafter. We measured these markers in long-distance runners before, immediately after, and several days after a race. We also compared these levels with those of people who do not regularly perform endurance exercise. We found that several of these markers increased after running, particularly in runners who showed signs of airway narrowing, which can make breathing harder. These changes mostly returned to normal after a few days. This suggests a heightened immune activation during and immediately after prolonged running that may play a role in temporary breathing difficulties. Understanding these changes could help identify athletes at risk and improve how we support respiratory health during intense physical activity. Our findings could lead to better training plans and recovery strategies for runners and other endurance athletes.

## 1. Introduction

Marathon running, an endurance sport covering 42.195 km (26.2 miles), exerts substantial physical stress on the body. While moderate physical activity is well-documented to prevent or improve conditions such as obesity, hypertension [1], diabetes [2], osteoporosis [3], cancer [4], and aging-related decline [5], prolonged intense exercise, such as marathon running, triggers significant physiological responses. These include the release of proinflammatory markers and the activation of acute inflammatory pathways [6,7,8,9,10,11,12,13]. Conversely, it may also induce temporary immunosuppression, increasing susceptibility to infections, particularly in the upper respiratory tract [10,11]. This phenomenon is addressed by the “open window” theory, which proposes that intense physical exertion temporarily weakens the immune system, creating a period of heightened vulnerability to pathogens [6,10,11].

Antimicrobial peptides (AMPs) constitute a fundamental component of the innate immune defense, serving as a primary defense against a wide range of pathogens, including bacteria, viruses, and fungi [11,14,15]. Secreted by epithelial cells within the respiratory tract, they serve as key sentinels against microbial invasion. Evidence suggests that exercise may modulate both the expression and activity of AMPs, potentially influencing the host’s susceptibility to respiratory infections at mucosal surfaces [16].

Among AMPs, human beta-defensin 2 (hBD-2) and lactoferrin are well-characterized for both their antimicrobial efficacy and immunomodulatory functions. hBD-2 contributes to host defense by targeting diverse pathogens and is also implicated in modulating immune responses and serving as a potential biomarker of inflammation [17]. In addition to their direct antimicrobial activity, hBD-2 and lactoferrin orchestrate various immune processes, including immune cell recruitment, regulation of inflammatory responses, and facilitation of tissue repair [18]. Recent research has increasingly underscored the pivotal role of AMPs in maintaining airway immune defense, particularly under conditions of physical stress such as exercise-induced bronchoconstriction (EIB) [8,11,19].

EIB is a condition characterized by the narrowing of the airways during or after exercise, manifesting as coughing, wheezing, shortness of breath, and chest tightness. It is especially prevalent in athletes and individuals engaged in high-intensity endurance sports such as running, swimming, and cycling [20,21,22]. EIB affects 5–20% of the general population, and in nonasthmatic individuals, exercise is the sole trigger. The prevalence ranges from 8–20%. Environmental factors, such as exposure to cold air, pollutants, or allergens, can exacerbate symptoms [23,24]. The pathophysiology of EIB involves the rapid inhalation of large volumes of air during exercise, which cools and dries the airways, leading to inflammation and airway hyperresponsiveness [24].

hBD-2 is secreted by various cell types in response to microorganisms or proinflammatory cytokines [17,25]. Beyond its antimicrobial activity, hBD-2 facilitates immune cell chemotaxis and activates Toll-like receptors (TLRs) [17,26]. In smokers, increased expression of beta-defensins in lung tissue highlights hBD-2′s role in protecting the respiratory tract from oxidative stress and infections linked to chronic obstructive pulmonary disease (COPD). Conversely, elevated hBD-2 and hBD-4 levels have been specifically detected in patients with mucoid *Pseudomonas aeruginosa* infections, correlating with serum C-reactive protein (CRP) levels [27,28]. Mutations in genes encoding hBD-2 (DEFB4A and DEFB4B) have been associated with atopic asthma in children [29,30]. Elevated levels of hBD-2 have also been observed in chronic respiratory infections and idiopathic pulmonary fibrosis, suggesting its potential as a biomarker for disease progression [27,31].

S100A8 and S100A9 are calcium-binding proteins of the S100 family that play a central role in inflammatory responses. They are predominantly expressed in myeloid-derived cells such as neutrophils and monocytes, where they typically form a heterodimeric complex known as S100A8/A9 [32,33,34,35,36,37]. Within this complex, S100A8 has been identified as the functionally more active component [38]. In addition to their well-established proinflammatory functions, S100A8/A9 exhibit antimicrobial properties and support immune cell recruitment, contributing significantly to innate immune defense [33]. Moreover, they enhance cytokine secretion in inflammatory cells [33]. Interestingly, S100A8 alone has been shown to suppress mast cell degranulation and inhibit the release of IL-4, IL-6, and GM-CSF in response to IgE crosslinking by reducing reactive oxygen species (ROS) production in vitro. In a mouse model of acute asthma, S100A8 attenuated mast cell degranulation, the production of eosinophil chemoattractants, and eosinophil infiltration [39]. It also negatively regulates leukocyte adhesion and transmigration by inhibiting p38 MAPK phosphorylation [38]. Consistently with its involvement in allergic airway inflammation, S100A8 expression was found to be significantly upregulated in the lungs of asthmatic rats [40]. Conversely, in a murine model of acute lung injury, S100A8 promoted inflammation through Toll-like receptor 4 (TLR4)-dependent activation of alveolar epithelial cells [41] highlighting its proinflammatory potential. These findings underscore the context-dependent nature of S100A8 activity, which can exert either protective or pathogenic effects depending on the immune environment and disease model.

S100A8/A9, also known as calprotectin, is upregulated in numerous inflammatory conditions, including asthma [42], diabetes [43], arthritis [44], and chronic inflammatory bowel disease [45]. This complex exhibits antimicrobial activity and promotes immune cell recruitment, playing a critical role in the innate immune response [33]. Elevated levels of S100A8/A9 have been linked to airway hyperresponsiveness in asthma. Studies have shown that serum S100A8/A9 levels correlate with lung function and airway hyperresponsiveness, suggesting that S100A8/A9 serves as a biomarker [39,42,46]. Experimental evidence suggests that inhibiting S100A8/A9 improves pathological conditions in murine models. For instance, S100A9 knockout mice, which lack the subunit that stabilizes the functional S100 protein heterodimer, exhibited attenuated hallmarks of asthma, including airway inflammation, airway hyperresponsiveness, and airway remodeling, in a murine model of allergic asthma [33,46].

MBP, a low-molecular-weight, highly cationic protein released by eosinophils, is implicated in airway mucosal injury and bronchial hyperresponsiveness. Eosinophils and their released proteins play a central role in allergic airway inflammation [47,48]. MBP has cytotoxic effects on helminths, bacteria, and mammalian cells and is found deposited on damaged lung epithelial tissue in asthma patients [49,50,51,52]. MBP also contributes to bronchial hyperreactivity and stimulates cytokine production in activated cells [53,54]. Research has indicated that sputum polymorphonuclear neutrophils (PMNs) were increased in nonasthmatic runners both after a marathon and at baseline, suggesting that exercise-associated inflammatory airway changes may involve eosinophil activation and MBP release [55].

Angiogenin, a member of the ribonuclease family, circulates freely in human plasma and exhibits a broad spectrum of functions, including wound healing and tissue repair [56]. It plays a key role in angiogenesis, particularly in malignant diseases, where it was first identified in carcinoma [57,58]. Angiogenesis, in response to tissue injury, is a dynamic process that is highly regulated by signals from both serum and the surrounding extracellular matrix environment [59]. Additionally, it acts as an acute-phase reactant [57,60,61]. For instance, Olson et al. [61] reported that angiogenin levels were elevated threefold in hospitalized patients compared with healthy controls. The acute phase response is a systemic reaction to disturbances in homeostasis caused by infection, tissue injury, trauma, or surgery. It is characterized by changes in the concentrations of plasma proteins, collectively known as acute-phase proteins. Exercise, particularly prolonged endurance activities such as marathon running, can induce an acute phase response [6,7,8,9,10,13,62]. Studies have shown that serum levels of acute-phase reactants increase following endurance exercise, demonstrating similarities between this response and the response consequent to general medical and surgical conditions [63]. In summary, angiogenin is a multifunctional protein involved in angiogenesis, wound healing, and tissue repair. Its role as an acute-phase reactant suggests that it may be involved in the body’s response to prolonged endurance activities, such as marathon running.

To investigate alterations in AMPs during extraordinary physical activity and their potential role in the development of EIB, we analyzed serum levels of AMPs in runners before, immediately after, and a few days following a marathon or half-marathon. These results were compared with those of healthy sedentary controls. Additionally, spirometric values were assessed to detect the occurrence of EIB. Our aim was to gain a deeper understanding of the systemic secretion of AMPs during physical stress and their potential contribution to EIB.

## 2. Material and Methods

### 2.1. Setting

For this study, male and female runners of a full or half-marathon (Vienna City Marathon, held on 5 April 2012) were included and assessed at three different timepoints: the day before (further referred to as baseline), immediately after (peak), and two to seven days after the marathon (recovery). The spirometry was performed using a portable lung function testing device (PC Spirometry, SDS 104, Schiller AG, Linz, Austria) to assess FVC%, FEV1%, MEF75, MEF50, MEF25, and the FEV1/FVC ratio at all three timepoints. On the day of the marathon, the mean air pressure was 974.8 hPA, the mean air temperature was 10.8 °C, the mean relative air humidity was 87.3%, the wind direction was northwest, the wind force was between 12 and 19 km/h, and there was no precipitation. Patients suffering from exercise-induced bronchoconstriction (EIB) were defined as having a decrease of ≥10% in FEV1 from baseline. None of the participants suffered from COPD, asthma, or any other condition that causes respiratory symptoms during or after physical exercise. There were no bronchodilators taken by participants or administered for study purposes.

Ethical approval was obtained from the institutional review board (EK 1034/2012) of the Medical University of Vienna. All tests were performed in accordance with the Declaration of Helsinki and the guidelines for good scientific practice of the Medical University of Vienna. All subjects participating in this study gave written informed consent.

#### 2.1.1. Laboratory Procedures

Blood samples were collected at baseline, peak, and recovery. Blood was taken from the antecubital vein in EDTA and serum gel tubes. EDTA tubes were used for blood count analysis using a hematology analyzer (Sysmex KX-21N, Sysmex Corporation, Kobe, Japan) and for investigations of routine parameters. Serum was separated by centrifugation (15 min at RCF 2845× *g*) and used for quantification of the indicated proteins. All samples were stored at −80 °C within 2 h after the event. The study was conducted at the research laboratories of the Department of Thoracic Surgery at the Medical University of Vienna.

#### 2.1.2. Detection of Proteins in Serum

Commercially available enzyme-linked immunosorbent assays (ELISA) were used to measure serum concentrations of the indicated antimicrobial proteins. These ELISAs were performed according to the manufacturers’ protocols. The employed quantitative immunoassays were purchased as follows: angiogenin (RnD Systems, Minneapolis, MN, USA), S100A8/A9 (RnD Systems, Minneapolis, MN, USA), S100A8 (RnD Systems, USA), human beta-defensin 2 (Elabscience, Houston, TX, USA), and major basic protein (MyBioSource, San Diego, CA, USA). Researchers performing the laboratory work and data analyses were blinded to the study groups associated with each sample.

#### 2.1.3. Statistical Methods

Data was analyzed using the SPSS software (version 29; IBM SPSS Inc., Chicago, IL, USA). To compare the means of two and more than two independent groups with normal distributions, unpaired Student’s *t*-tests and one-way ANOVA were applied, respectively. For non-normal distributions, the Kruskal–Wallis rank test was used. The specific tests used are indicated in the table and/or results section. Post hoc comparisons were conducted using Bonferroni correction. For comparison of repeated measurements in the same study, the subject paired t-test was used in normally distributed data. For non-normally distributed data, the Wilcoxon test was used. Non-normally distributed data are presented as median and interquartile range. To assess linear relationships between two numerical variables, Pearson’s product-moment correlation was employed, reported as the Pearson correlation coefficient (r). Figures were generated using GraphPad Prism 9 (GraphPad Software Inc., La Jolla, CA, USA).

## 3. Results

### 3.1. Demographic and Spirometry Data

In this study, the serum and data of 34 marathoners, 36 half-marathoners, and 30 sedentary volunteers were collected. Detailed demographic data, spirometry data, and running and training history can be found in previously published studies [62,64] and are depicted in summarized form in Table 1 (adapted from Bekos et al. [62,64]).

Spirometry data and serum levels of inflammatory markers taken before the run, immediately after the run, and 2–7 days after the run are depicted in Table 2 (adapted from Bekos et al. [62,64]).

### 3.2. Serum Measurement of Circulating Antimicrobial Peptides

#### 3.2.1. Calprotectin

**Figure 1:**
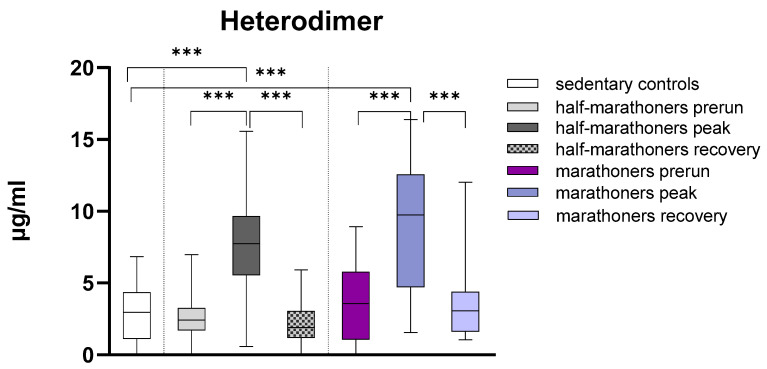
Serum concentrations of S100A8/S100A9 (µg/mL) in marathoners and half-marathoners at baseline, peak, and recovery, as well as in sedentary controls. Baseline, 1–2 days before the run; peak, immediately after the run in the finishing area; recovery, after 2–7 days of recovery; *** *p* < 0.001.

Calprotectin was significantly elevated in half-marathoners and marathoners reaching the finishing areas (*p* < 0.001) compared with baseline serum values and sedentary controls. After peaking in the finishing area, calprotectin returned to baseline values 7 days after running. There was no significant difference in calprotectin levels between half-marathoners and marathoners (Figure 1).

#### 3.2.2. Angiogenin

**Figure 2:**
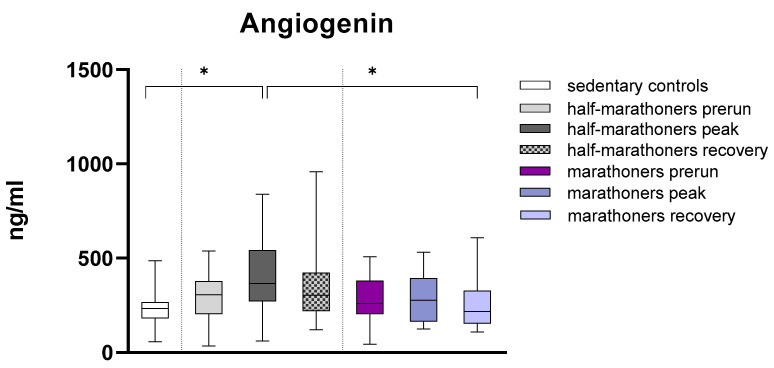
Serum concentrations of angiogenin (ng/mL) in marathoners and half-marathoners at baseline, peak, and recovery, as well as in sedentary controls. Baseline, 1–2 days before the run; peak, immediately after the run in the finishing area; recovery, after 2–7 days of recovery; * *p* < 0.05.

Angiogenin was significantly elevated in half-marathoners immediately after running (365.4 ng/mL) compared with sedentary controls (232.6 ng/mL, *p* = 0.038) and marathoners 7 days after running (217.8 ng/mL, *p* = 0.028) (Figure 2). Angiogenin levels in marathoners remained largely unchanged when comparing prerun, peak, and postrun measurements. In half-marathoners, angiogenin levels showed larger differences when comparing prerun and postrun measurements with peak measurements, although these differences were not statistically significant. Medians were compared using Kruskal–Wallis tests and Bonferroni post hoc correction for multiple comparisons.

#### 3.2.3. S100A8

**Figure 3:**
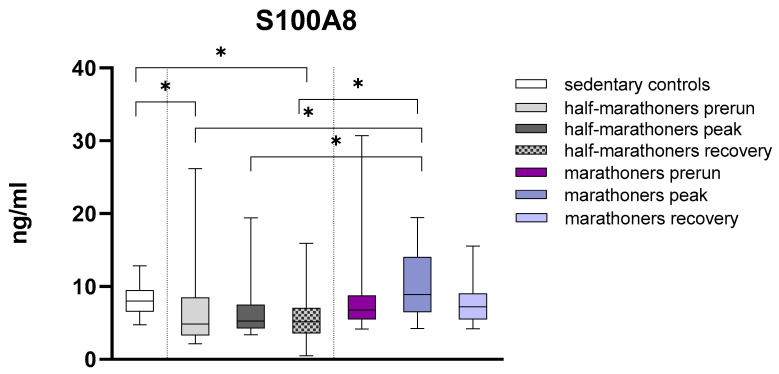
Serum concentrations of S100A8 (ng/mL) in marathoners and half-marathoners at baseline, peak, and recovery, as well as in sedentary controls. Baseline, 1–2 days before the run; peak, immediately after the run in the finishing area; recovery, after 2–7 days of recovery; * *p* < 0.05.

Serum values of S100A8 were significantly reduced in half-marathoners at baseline (*p* = 0.028) and recovery (*p* = 0.008) compared with sedentary controls. S100A8 was conversely significantly elevated in marathoners reaching the finishing area compared with all timepoints (all *p* < 0.01) in half-marathoners. Albeit not in a statistically significant way, marathoners exhibited elevated recovery values compared with half-marathoners (*p* = 0.06) (Figure 3).

#### 3.2.4. Major Basic Protein

**Figure 4:**
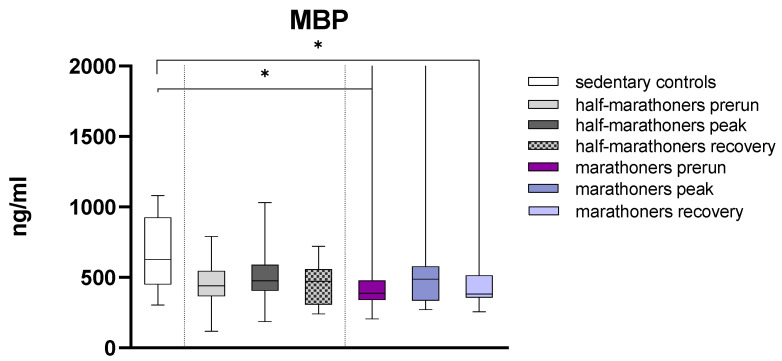
Serum concentrations of major basic protein (ng/mL) in marathoners and half-marathoners at baseline, peak, and recovery, as well as in sedentary controls. Baseline, 1–2 days before the run; peak, immediately after the run in the finishing area; recovery, after 2–7 days of recovery; * *p* < 0.05.

Major basic protein was significantly higher in sedentary controls compared with baseline and recovery serum levels of marathoners (627 ng/mL, IQR: 450.2–926.0 vs. 388 ng/mL, IQR: 341.1–477.2 and 382.9, IQR: 357.2–514.3, *p* = 0.035 and *p* = 0.014) (Figure 4).

#### 3.2.5. Human Beta-Defensin 2

**Figure 5:**
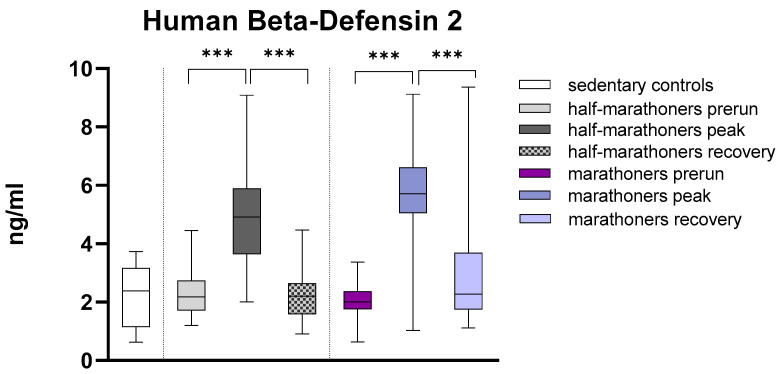
Serum concentrations of human beta-defensin 2 (ng/mL) in marathoners and half-marathoners at baseline, peak, and recovery, as well as in sedentary controls. Baseline, 1–2 days before the run; peak, immediately after the run in the finishing area; recovery, after 2–7 days of recovery; *** *p* < 0.001.

Human beta-defensin 2 was highly significant elevated in half-marathoners (4.91 ng, IQR: 3.6–5.9) and marathoners (5.7 ng, IQR: 5.1–6.6) reaching the finishing area compared to their baseline values (HM: 2.18, IQR: 1.72–2.74 and M: 2.02, IQR: 1.77–2.37) and sedentary controls (Figure 5). After 2–7 days of recovery the value returned to baseline. The detailed results of Kruskal–Wallis analysis are listed below in Table 1.

To assess overall differences in marker levels across all study groups and timepoints, a Kruskal–Wallis test was performed. Detailed results with *p*-values of this analysis are given in Table 3. The sedentary controls, half-marathoners, and marathoners at baseline, immediately postrun, and during recovery were included.

### 3.3. EIB

Two to seven days postrun, there were slightly elevated levels of S100A8 (7025.86 ng/mL, ±595.31 vs. 9674.48 ng/mL, ±1142.12; *p* = 0.053) in marathoners with EIB. Similarly, there were slightly elevated levels of human beta-defensin 2 (2.25 ng/mL, ±0.17 vs. 3.2, ±0.5; *p* = 0.084) in the runners with EIB (HM + M) two to seven days postrun. Immediately after the run in the finishing area, there were slightly reduced angiogenin values in half- and marathoners with EIB (277.86 ng/mL, ±33.73 vs. 370.32 ng/mL, ±25.62, *p* = 0.058).

There was a negative correlation between MEF75 and S100A8 in half-marathoners and marathoners (r = −0.7; *p* = 0.002) and TIFF (−0.53; *p* = 0.029). Human beta-defensin 2 was positively correlated with MBP (r = 0.59; *p* = 0.02).

In half-marathoners, there were strong correlations between heterodimer and MBP (r = 0.9; *p* = 0.002) and MEF25 (r = 0.86; *p* = 0.013). Between MBP and MEF25, there was also a positive correlation (r = 0.853, *p* = 0.015).

**Figure 6:**
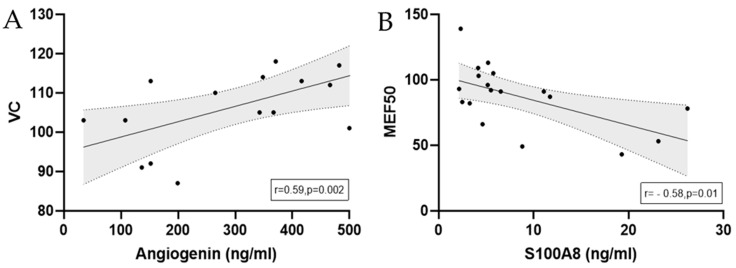
Correlations with regression lines of baseline serum concentrations of (**A**) angiogenin and (**B**) S100A8 and spirometry results in marathoners and half-marathoners. Correlations were calculated using Pearson correlation. Baseline, 1–2 days before the run.

Significant correlations between calculated antimicrobial peptide levels and spirometry data of marathoners and half-marathoners at baseline are depicted in Figure 6. 

**Figure 7:**
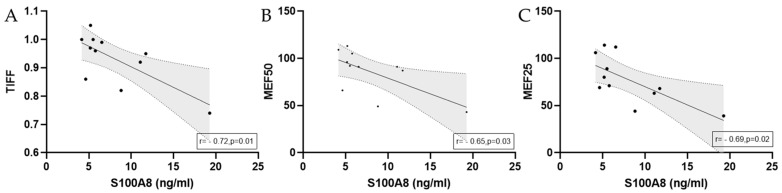
Significant correlations with regression lines of baseline serum concentrations between S100A8 and (**A**) TIFF, (**B**) MEF50, and (**C**) MEF25 in marathoners. Correlations were calculated using Pearson correlation. Baseline, 1–2 days before the run; TIFF, Tiffeneau–Pinelli index; MEF50, maximal expiratory flow at 50% of forced vital capacity; MEF25, maximal expiratory flow at 25% of forced vital capacity.

Significant correlations between calculated antimicrobial peptide levels and spirometry data of marathoners at baseline are depicted in Figure 7.

There were no correlations in the group of half-marathoners between baseline serum levels of antimicrobial peptides and spirometry outcomes.

**Figure 8:**
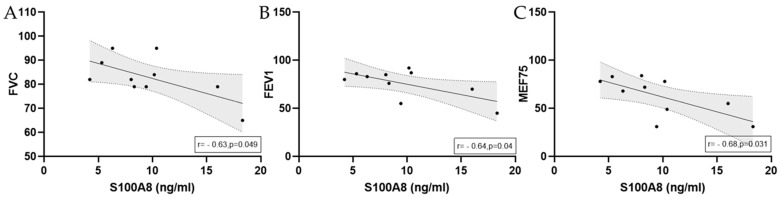
Significant correlations with regression lines of peak serum concentrations between S100A8 and (**A**) FVC, (**B**) FEV1, and (**C**) MEF75 in marathoners. Correlations were calculated using Pearson correlation. Peak, immediately after the run; FVC, forced vital capacity; FEV1, forced expiratory volume in 1 s; MEF75, maximal expiratory flow at 75% of FVC.

In marathoners reaching the finishing area and showing signs of exercise-induced bronchoconstriction, there were significant negative correlations between S100A8 serum levels and FVC, FEV1, and MEF75. Graphical illustrations with linear regression lines are depicted in Figure 8.

**Figure 9:**
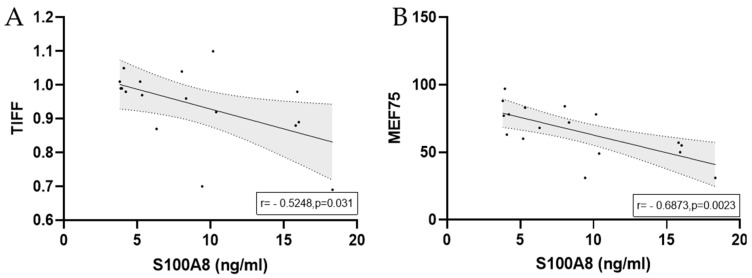
Significant correlations with regression lines between peak serum concentrations of S100A8 and (**A**) TIFF and (**B**) MEF75 in marathoners and half-marathoners. Correlations were calculated using Pearson correlation. Peak, immediately after the run; TIFF, Tiffeneau–Pinelli index; MEF75, maximal expiratory flow at 75% of FVC.

When considering all runners with EIB at the finishing area, there were significant negative correlations between S100A8 serum levels and TIFF and MEF75 (Figure 9).

#### Correlations of Cytokines and Calculated Antimicrobial Peptides in Half-Marathoners and Marathoners with EIB

The following table (Table 4 summarizes significant correlations between serum levels of cytokines and antimicrobial peptides in marathon and half-marathon runners, assessed at three timepoints: baseline (1–2 days before the race), peak (immediately postrace), and recovery (2–7 days after the race). Correlation analysis highlighted associations between immune-related markers such as S100A8/A9, ST2, esRAGE, and MBP. A correlation analysis of AMPs with additional laboratory parameters is available in the Supplementary data.

## 4. Discussion

This study suggests that marathon and half-marathon running substantially influence immune and inflammatory markers, as evidenced by elevations in antimicrobial peptides (AMPs) and related inflammatory markers. Notably, human beta-defensin 2 (hBD-2), a central AMP, was significantly increased in marathon runners immediately after race completion, indicating transient immune activation. Elevated levels of hBD-2 may enhance mucosal defenses against pathogens—a critical function during the postexercise “open window” period, characterized by temporary immunosuppression and heightened infection susceptibility in endurance athletes [65]. hBD-2 has been previously associated with protective airway functions, especially in asthma and chronic airway inflammation [17,30]. This supports its potential role in mitigating bronchoconstriction and infection following strenuous physical exertion.

Similarly, S100A8/A9 (calprotectin) showed a marked postexercise increase followed by a return to baseline during recovery, reflecting its role as a damage-associated molecular pattern (DAMP) and a marker of systemic inflammation triggered by both physical stress and chronic inflammatory airway diseases [33,66]. As a DAMP, calprotectin activates Toll-like receptor 4 (TLR4) or the receptor for advanced glycation end products (RAGE), thereby initiating proinflammatory signaling cascades involving nuclear factor κB (NF-κB) and mitogen-activated protein kinases (MAPKs) [67,68,69,70]. These pathways are known to mediate airway inflammation and asthma-associated airway hyperresponsiveness [33,37,39,71,72]. These pathways are central to airway inflammation and asthma-related hyperresponsiveness. Importantly, antimicrobial peptides (AMPs) such as hBD-2 and S100A8/A9 are not only relevant to exercise-induced bronchoconstriction (EIB) but implicated in other obstructive airway diseases, including asthma and allergic inflammation. hBD-2 expression is upregulated not only in response to pathogens but during allergen-induced inflammation, where it contributes to epithelial barrier regulation and Th2-type immune responses [17,30]. In murine models of allergic asthma, deletion or inhibition of S100A8 or S100A9 has been shown to reduce airway inflammation, eosinophilic infiltration, and structural remodeling [46]. The observed inverse relationship between S100A8 levels and lung function in EIB-positive athletes may therefore reflect epithelial activation via TLR4 signaling in response to mechanical or immune stress during prolonged exercise. This is supported by previous findings showing that S100A8 triggers IL-6 and MCP-1 production and neutrophil recruitment through TLR4 activation in alveolar epithelial cells, exacerbating inflammation in models of acute lung injury [41,73]. A similar mechanism may contribute to airway narrowing and impaired lung function in EIB.

In contrast, levels of major basic protein (MBP), a cytotoxic eosinophil-derived molecule, were higher in sedentary controls than in athletes. This suggests that regular endurance training is associated with reduced basal eosinophilic activation [74].

Angiogenin is a protein involved in vascular remodeling that also functions as an acute-phase reactant [57,61]. In EIB-positive athletes (HM + M), it was positively correlated with lung function at baseline but decreased significantly postrace. This seemingly contradictory pattern may reflect transient vascular and immune dysregulation under acute bronchoconstrictive stress. The positive baseline association suggests a protective vascular or anti-inflammatory role in well-trained individuals, as supported by previous reports [75,76]. In addition, hypoxia-induced angiogenin expression indicates its involvement in broader vascular stress responses [77,78,79]. Its decline after exercise in EIB-positive runners may thus represent acute consumption or depletion of physiological reserves. Similar transient decreases have been observed for other acute-phase proteins, where rapid utilization contributes to immune vulnerability [7,8,9,63,80,81]. For example, Santos et al. [9] reported increased DNA fragmentation and enhanced expression of L-selectin and Fas receptors during the recovery period after a marathon, indicating delayed neutrophil activation and apoptosis consistent with the “open window” hypothesis [9].

When considering both EIB-positive and EIB-negative athletes together, angiogenin levels displayed a race-type-specific pattern: postrun elevations were significant in half-marathon runners compared with sedentary controls, while marathon runners showed stable levels throughout. This contrast supports the notion that shorter, high-intensity endurance efforts trigger more pronounced but transient inflammatory responses than prolonged submaximal exertion [10,13,82]. Legaz-Arrese et al. further demonstrated that exercise intensity has a greater influence on cardiac biomarkers such as troponin I than total exercise volume [83]. In summary, angiogenin may act both as a protective and a consumption-sensitive acute-phase protein. Its functional role likely varies with the degree and type of physiological or inflammatory challenge, being mobilized under moderate stress and depleted under severe bronchoconstrictive strain.

At baseline, rather than indicating simply elevated or suppressed levels, the strong positive correlation between ST2 and angiogenin observed in both groups (HM: *r* = 0.70, *p* = 0.002; M: *r* = 0.84, *p* = 0.004) may reflect a more balanced and responsive regulation of vascular stress signaling in endurance-trained individuals. This aligns with previous findings showing that regular training prevents excessive or pathological ST2 elevations under stress [84]. ST2 has also been associated with oxidative and inflammatory markers such as intracellular adhesion molecule-1 (ICAM-1) and IL-6 in various disease contexts, supporting its role in vascular inflammation [85]. Similarly, the baseline associations among S100A8/A9, esRAGE, MBP, and CRP likely reflect a dynamic yet controlled inflammatory–oxidative axis. Supporting this, a military endurance training intervention reported significant reductions in both S100A8/A9 and sRAGE within just four weeks, suggesting adaptive modulation of immune–oxidative pathways in response to consistent training stimuli [86].

During peak exercise, the strong correlation between S100A8/A9 and MBP in half-marathoners (*r* = 0.90, *p* = 0.006) points to synchronized neutrophilic and eosinophilic activation under acute stress—reminiscent of the mixed inflammation phenotype observed in certain asthma subtypes [87,88]. Among marathon participants, the positive correlation between S100A8/A9 and IL-33 (*r* = 0.79, *p* = 0.01) is consistent with the known role of S100A8/A9 as an immune alarmin, capable of triggering cytokine release via epithelial signaling pathways [41,89]. In contrast, the observed negative correlation between S100A8 and AGE-CML (*r* = −0.64, *p* = 0.05) mirrors findings from inflammatory disease contexts, where elevated S100A8 levels are inversely associated with sRAGE concentrations [90].

During recovery, the strong correlation between angiogenin and hBD-2 in half-marathoners (*r* ≈ 0.84–0.85) suggests coordinated vascular and mucosal immune repair processes. This is in line with systematic evidence of exercise-induced angiogenic shifts, including VEGF-mediated capillary remodeling following endurance exertion [91]. Meanwhile, the sustained correlation between MBP and esRAGE in marathon runners (*r* = 0.84, *p* = 0.003) points to ongoing eosinophilic activation intertwined with oxidative stress during the postexercise phase. This supports findings from endurance studies showing that reactive oxygen species (ROS) and oxidative damage markers can remain elevated for up to 48 h after exercise, despite concurrent activation of antioxidant defenses [92,93]. Moreover, eosinophil-derived cationic proteins have been directly linked to oxidative stress in inflammatory environments, suggesting that eosinophil granule release may itself contribute to redox signaling during recovery [94,95].

Most existing studies on AMP dynamics during exercise have focused on local changes in mucosal or salivary compartments [16,96,97,98,99]. In contrast, our data demonstrates that circulating AMPs fluctuate systemically in response to endurance activity and correlate with respiratory function and EIB susceptibility. These systemic shifts differed between athletes and sedentary controls at both baseline and postexercise, suggesting long-term immune adaptation in trained individuals. While mucosal AMP expression provides important insights into local immune defense, circulating AMPs may better capture whole-body immune dynamics. This could prove particularly valuable for identifying athletes at risk of systemic immune dysregulation during the postexercise recovery period.

### Limitations

This study has several limitations. First, although the sample size was sufficient to detect moderate effect sizes, it may constrain the generalizability of the findings to wider athletic populations. All participants were drawn from a single event under specific environmental conditions, limiting extrapolation to different climates or race settings.

Second, the observational study design and time-point-based sampling preclude definitive conclusions regarding causality. Although we observed correlations between AMP concentrations and pulmonary function, longitudinal analyses would be required to determine the predictive or mechanistic role of AMPs in EIB pathogenesis.

Third, while participants with pre-existing respiratory diagnoses were excluded, the possibility of subclinical airway conditions cannot be entirely excluded. Furthermore, the diagnosis of EIB was based solely on a ≥10% decline in FEV1 without confirmatory bronchial provocation testing, potentially introducing misclassification.

Fourth, the lack of detailed data on training volume adjustments in the final days before the race—such as tapering or overload—represents a potential confounding factor.

Despite these limitations, our findings provide novel insights into the temporal dynamics of systemic AMPs during endurance exercise and their potential role in EIB.

## 5. Conclusions

This study demonstrates that marathon and half-marathon running elicits marked systemic immune and inflammatory responses, particularly involving antimicrobial peptides (AMPs) such as hBD-2 and S100A8/A9. Notably, hBD-2 increased immediately postrace, suggesting a role in enhancing mucosal defense during the transient immunosuppressive “open window” phase. S100A8 and its association with airway inflammation through TLR4 signaling further underline its potential mechanistic involvement in EIB. Angiogenin dynamics revealed a dual role, with baseline levels correlating positively with lung function and postrace declines in EIB-positive athletes suggesting functional depletion under bronchoconstrictive stress. Correlation analyses between immune and oxidative stress markers (e.g., MBP and esRAGE) point toward a coordinated immuno-redox response to exertional strain. Together, these findings highlight the relevance of systemic AMPs and related mediators as dynamic biomarkers for immune modulation in endurance athletes and provide new insights into the potential pathophysiological underpinnings of EIB.

## Figures and Tables

**Table 1:** Demographic and training details of the study cohort. Data are given with median and interquartile range.

	MARATHON (*N* = 34)	HALF-MARATHON (*N* = 36)	CONTROLS (*N* = 30)
AGE (YEARS)	36.5 (32–44)	37.0 (30.00–42.00)	29.00 (25.00–41.00)
F:M (QUANTITY)	10:24	17:19	13:17
BMI (KG/M2)	22.51 (20.93–24,13)	23.47 (20.93–25.23)	22.99 (20.86–27.08)
WEIGHT (KG)	71.00 (63.20–76.40)	71.85 (58.60–86.35)	73.00 (64.45–83.75)
BODY FAT PERCENTAGE (%)	17.40 (12.80–23.00)	24.45 (20.85–30.33)	21.45 (16.03–27.40)
TARGET TIME (MIN)	230.00 (201.75–251.75)	112.00 (106.00–128.50)	0
KM PER WEEK	60.00 (40.00–80.00)	35.00 (30.00–40.00)	0
TRAINING DAYS/WEEK	4.00 (4.00–5.00)	3.00 (3.00–4.00)	0

**Table 2:** Spirometry data and investigated serum levels of CRP, C-reactive protein; IL33, interleukin-33; AGE-CML, advanced glycation end products–carboxymethyllysine; esRAGE, endogenous secreted receptor for advanced glycation end products; HM, half-marathon; M, marathon; FVC, forced vital capacity; TIFF, Tiffeneau–Pinelli index; MEF75, maximal expiratory flow at 75% of forced vital capacity; MEF50, maximal expiratory flow at 50% of forced vital capacity; MEF25, maximal expiratory flow at 25% of forced vital capacity. Data are given in median and IQR. Adapted from Bekos et al. [62,64].

	Sedentary Controls	HM Baseline	HM Peak	HM Recovery	M Baseline	M Peak	M Recovery
FVC	94.5 (85.00–105.25)	100.00 (90.00–105.00)	93.00 (80.00–101.50)	94.00 (85.00–103.00)	99.50 (94.00–105.00)	87.00 (79.00–95.50)	102.00 (90.00–108.75)
FEV1	90.50 (79.00–101.00)	96.00 (88.00–101.00)	88.00 (81.50–96.50)	91.00 (83.00–101.00)	95.50 (90.00–102.00)	87.50 (81.00–95.25)	96.50 (87.00–101.00)
VC	91.50 (82.00–100.50)	101.00 (87.00–110)	93.00 (83.50–102.00)	99.00 (91.00–105.00)	97.50 (87.25–107.50)	87.00 (78.00–93.00)	99.00 (89.00–114.00)
TIFF	0.97 (0.90–1.02)	0.97 (0.94–0.99)	0.97 (0.93–1.01)	0.96 (0.92–0.99)	0.97 (0.92–1.00)	0.98 (0.92–1.07)	0.96 (0.90–0.99)
MEF75%	85.00 (68.75–96.00)	84.00 (71.00–99.5)	80.00 (67.50–88.00)		86.00 (76.50–102.00)	80.00 (68.00–89.75)	
MEF50%	89.00 (64.75–103.00)	86.50 (74.00–103.75)	83.00 (67.00–90.50)		91.00 (76.00–105.00)	80.50 (66.25–94.50)	
MEF25%	81.00 (55.25–106.50)	86.00 (69.00–109.00)	82.00 (65.00–105.00)		82.00 (68.00–107.00)	82.50 (67.50–105.00)	
CRP (mg/dL)	0.11 (0.00–0.28)	0.05 (0.00–0.16)	0.05 (0.00–0.14)	0.10 (0.05–0.23)	0.00 (0.00–0.07)	0.00 (0.00–0.053)	0.13 (0.08–0.25)
IL33 (pg/mL)	44.81 (16.25–292.85)	64.02 (0.00–507.88)	77.37 (0.00–483.78)	71.52 (0.00–335.42)	58.28 (0.00–562.75)	86.18 (15.93–638.16)	66.30 (0.00–439.65)
AGE-CML (ng/mL)	33.37 (24.71–42.52)	52.73 (39.86–61.31)	61.77 (53.31–70.96)	51.83 (44.79–64.31)	34.46 (26.88–48.56)	41.91 (36.45–53.50)	34.70 (28.76–44.08)
esRAGE (ng/mL)	0.32 (0.23–0.43)	0.34 (0.24–0.44)	0.48 (0.36–0.60)	0.38 (0.27–0.51)	0.31 (0.25–0.37)	0.36 (0.28–0.51)	0.29 (0.21–0.38)

**Table 3:** Serum concentrations of antimicrobial peptides in marathoners and half-marathoners at baseline, peak, and recovery and sedentary controls. Data are given with median and interquartile ranges. Timepoints and groups were compared using Kruskal–Wallis with Bonferroni corrections for multiple testing. HM, half-marathoners; M, marathoners; baseline, 1–2 days before the run; peak, immediately after the run in the finishing area; recovery, after 2–7 days of recovery.

	Sedentary Controls	HM Baseline	HM Peak	HM Recovery	M Baseline	M Peak	M Recovery	*p*-Value
Angiogenin (ng/mL)	233 (182–267)	306 (204–379)	365 (272–542)	303 (221–424)	260 (204–381)	277 (164–393)	218 (155–328)	0.015
S100A8/A9 (µg/mL)	2.96 (1.12–4.36)	2.43 (1.71–3.26)	7.75 (5.56–9.66)	3.04 (1.20–3.04)	3.58 (1.05–5.78)	9.75 (4.72–12.56)	9.75 (4.72–12.56)	<0.001
S100A8 (ng/mL)	8.02 (6.56–9.49)	4.85 (3.31–8478)	5.25 (4.24–7.49)	5.16 (3.59–7.04)	6.80 (5.50–8.75)	8.89 (6.48–14.04)	7.23 (5.49–9.03)	<0.001
Major Basic Protein (ng/mL)	627 (450–926)	440 (368–547)	476 (405–589)	472 (307–558)	388 (341–477)	488 (335–578)	383 (357–514)	0.022
Human Beta-Defensin 2 (ng/mL)	2.38 (1.15–3.16)	2.18 (1.72–2.74)	4.91(3.64–5.89)	2.21 (1.60–2.64)	2.02 (1.77–2.37)	5.72 (5.10–6.61)	2.27 (1.75–3.69)	<0.001

**Table 4:** Correlations of serum concentrations of antimicrobial peptides with cytokines in marathoners and half-marathoners at baseline, peak, and recovery. Data are given with correlation coefficients (R). Values were correlated using the Pearson correlation coefficient. HM, half-marathoners; M, marathoners; CRP; C-reactive peptide; esRAGE, endogenous secreted receptor for advanced glycation end-products; MBP, major basic protein; AGECML, advanced glycation end products–carboxymethyllysine, HBDF2, human beta-defensin 2; ST2, suppression of tumorigenicity 2; baseline, 1–2 days before the run; peak, immediately after the run in the finishing area; recovery, after 2–7 days of recovery.

Group	Subgroup	Variables Correlated	Correlation Coefficient (r)	*p*-Value
Baseline	HM	ST2 ↔ Angiogenin	0.70	0.002
Baseline	M	ST2 ↔ Angiogenin	0.84	0.004
Baseline	HM	S100A8 ↔ esRAGE	0.89	0.007
Baseline	M	MBP ↔ esRAGE	0.95	<0.001
Baseline	M	S100A8 ↔ CRP	0.68	0.02
Peak	HM	S100A8/A9 ↔ MBP	0.90	0.006
Peak	M	S100A8/A9 ↔ IL33	0.79	0.01
Peak	M	S100A8 ↔ AGECML	−0.64	0.05
Recovery	HM	Angiogenin ↔ HBDF2	0.84	0.02
Recovery	M	MBP ↔ esRAGE	0.84	0.003
Recovery	HM	Angiogenin ↔ HBDF2	0.85	0.032

## Data Availability

The raw data supporting the conclusions of this article will be made available by the authors on request.

## Supplementary Materials

The following supporting information can be downloaded at: https://www.mdpi.com/article/10.3390/biology14070825/s1, Table S1. Correlations of serum concentrations of antimicrobial peptides with other laboratory parameters in marathoners and half-marathoners at baseline, peak, and recovery.

## Abbreviations

The following abbreviations are used in this manuscript:

AGECML	Advanced glycation end products–carboxymethyllysine
AMP	Antimicrobial peptide
ARDS	Acute respiratory distress syndrome
CRP	C-reactive protein
DAMP	Damage-associated molecular pattern
EIB	Exercise-induced bronchoconstriction
ELISA	Enzyme-linked immunosorbent assay
FEV1	Forced expiratory volume in one second
FVC	Forced vital capacity
HBDF2/hBD-2	Human beta-defensin 2
HCT	Hematocrit
HM	Half-marathoner(s)
HMGB1	High mobility group box 1
ICAM-1	Intercellular adhesion molecule 1
ICU	Intensive care unit
IL-1RA	Interleukin 1 receptor antagonist
IL-6	Interleukin 6
IL-33	Interleukin 33
IQR	Interquartile range
MAPK	Mitogen-activated protein kinase
MBP	Major basic protein
MEF25/50/75	Maximal expiratory flow at 25%/50%/75% of FVC
MPV	Mean platelet volume
MPO	Myeloperoxidase
MXD	Mixed cell percentage
NE	Neutrophil elastase
NF-κB	Nuclear factor kappa B
PDW	Platelet distribution width
PLT	Platelet count
RAGE	Receptor for advanced glycation end products
ROS	Reactive oxygen species
r	Correlation coefficient
SD	Standard deviation
sRAGE	Soluble receptor for advanced glycation end products
ST2	Suppression of tumorigenicity 2
Th2	T-helper cell type 2
TIFF	Tiffeneau–Pinelli index (FEV1/FVC ratio)
TLR4	Toll-like receptor 4
VEGF	Vascular endothelial growth factor
WBC	White blood cell count
