# Supplementary material for: Circulating Antimicrobial Peptides as Biomarkers of Inflammation and Airway Dysfunction After Marathon Running
Source: Biology (Basel). 2025 Jul 7;14(7):825. doi: 10.3390/biology14070825 (PMC12292263; doi:10.3390/biology14070825)
Supplement: Supplementary file 1 [file biology-14-00825-s001.zip › biology-3681239-supplementary.pdf]

Supplementary Data

Correlations of antimicrobial peptides with other laboratory parameters

Half-Marathoners baseline

There was a correlation of Angiogenin with HMGB1 (r=0.38, p=0.03), HMGB1_HCT (r=0.39, p=0.02), HSP70 (r=0.45, p=0.01) and ST2 (r=0.40, p=0.02) baseline levels in half-marathoners. There was also a correlation between S100A8/S100A9 heterodimer with HSP70 (r=0.43, p=0.01) and ST2 (r=0.45, p=0.01). S100A8 serum levels correlated with sRAGE_feNa (r=0.054, p=0.004), IL33 (r=0.63, p<0.001), M65_urine (r=0.44, p=0.03), MCH (r=-0.36, p=0.04), MCHC (r=-0.36, p=0.04), PLT (r=-0.42, p=0.02), PDW (r=0.48, p=0.004), MPV (r=0.47, p=0.01) and P_LCR (r=0.44, p=0.01). There was also a correlation between HBDF2 serum levels and esRAGE (r=0.48, p=0.004), esRAGE_HCT (r=0.45, p=0.01), M30 Serum (r=0.43, p=0.03), M30 urin (r=-0.45, p=0.03) and fraktcCCK18 (r=-0.46, p=0.03).

Marathoners baseline

In marathoners there was a correlation between Angiogenin and PDW (r=-0.40, p=0.05). There was also a correlation between S100A8/A9 and HMGB1 (r=0.45, p=0.02), HMGB1_HCT (r=0.48, p=0.01), HSP27 (r=0.49, p=0.01), MXD procent (r=-0.49, p=0.02) and lactate (r=0.49, p=0.03). There was also a strong correlation between MBP and esRAGE (r=0.91, p<0.001) and esRAGE_HCT (r=0.92, p<0.001). There was also a correlation between HBDF2 and Neutr_procent (r=0.52, p=0.02).

Half-Marathoners peak

In half-marathoners there was a correlation of Angiogenin and S100A8 (r=0.37, p=0.03), HSP70 (r=0.36, p=0.04), RBC (r=0.37, p=0.03), HGB (r=0.43, p=0.01), HGBc (r=0.43, p=0.01), HCT (r=0.38, p=0.03), MCHCc (r=0.42, p=0.02) and MXD (r=0.34, p=0.05). There was also a correlation between S100A8/A9 and PLT (r=0.39, p=0.02) and MPVc (r=-0.36, p=0.042). In half-marathoners there was a correlation between S100A8 and Angiogenin and HBDF2 (r=0.34, p=0.05). There was also a correlation between MBP and HMGB1c (r=-0.42, p=0.03). There was also a correlation between HBDF2 and M30urin (r=0.62, p=0.001).

Marathoners peak

In marathoners there was a correlation between S100A8/A9 and AGECML (r=-0.42, p=0.034), AGECMLc (r=-0.48, p=0.012), AGECML_HCT (r=-0.42, p=0.03), HSP70 (r=0.48, p=0.012), fraktCK18 (r=0.45, p=0.02), LYM (r=-0.43, p=0.03). There was also a correlation of S100A8 and frakt cCK18 (r=0.42, p=0.04), frakt CCK18c (r=0.40, p=0.05). There was

also a correlation between MBP and esRAGE (r=0.80, p<0.001), esRAGEc (r=0.87, p<0.001), esRAGE_HCT (r=0.78, p<0.001). There was also a correlation between HBDF2 and M65M30urin (r=0.54, p=0.02), MEF75 (r=0.56, p=0.01), MEF50 (r=0.48, p=0.02), MEF25 (r=0.55, p=0.01).

Half-Marathoners recovery

In half-marathoners there was a correlation of Angiogenin and S100A8/A9 (r=0.42, p=0.01), HMGB1 (r=0.37, p=0.03), HMGB1_HCT (r=0.39, p=0.03), ST2 (r=0.50, p=0.01), WBC (r=0.35, p=0.04), MXD (r=0.46, p=0.01). There was also a correlation between S100A8/A9 and Angiogenin (r=0.42, p=0.01) and HBDF2 (r=0.36, p=0.04), HMGB1_HCT (r=0.35, p=0.05), ST2 (r=0.49, p=0.01), WBC (r=0.47, p=0.01), RBC (r=0.40, p=0.02), HGB (r=0.41, p=0.02), HCT (r=0.40, p=0.02), NEUT (r=0.48, p=0.004). There was also a correlation between S100A8 and ST2 (r=-0.51, p=0.004), RDW_CV (r=-0.43, p=0.013). There was also a correlation between MBP and HSP70 (r=0.36, p=0.04), IL1RA (r=-0.39, p=0.03) and PLT (r=0.41, p=0.02). There was also a correlation between HBDF2 and M65M30urin (r=0.60, p=0.01).

Marathoners recovery

In marathoners there was a correlation between Angiogenin and M65M30urin (r=0.52, p=0.03) and MCV (r=-0.46, p=0.03). There was also a correlation of S100A8/A9 and HMGB1 (r=0.41, p=0.04), HMGB1_HCT (r=0.41, p=0.04), RDW_SD (r=0.48, p=0.01), PDW (r=0.50, p=0.01), MPV (r=0.56, p=0.003) and P_LCR (r=0.54, p=0.004). There was also a correlation between S100A8 and sRAGE_HCT (r=-0.45, p=0.02), WBC (r=0.44, p=0.02), NEUT (r=0.42, p=0.04). There was also a correlation between MBP and esRAGE (r=0.66, p<0.01), esRAGE_HCT (r=0.63, p<0.001), FVC (r=-0.59, p=0.002), FEV1 (r=-0.40, p=0.04), TIFF (r=0.50, p=0.01), MEF25 (r=0.77, p=0.03), WBC (r=0.43, p=0.03) and NEUT (r=0.42, p=0.04). There was also a correlation between HBDF2 and CRP (r=0.44, p=0.04).

Table S1.

Correlations of Antimicrobial Peptides with Laboratory Parameters

Group	Subgroup	Variables Correlated	Correlation Coefficient (r)	p-value
Baseline	HM	Angiogenin ↔ HMGB1	0.39	0.02
Baseline	HM	Angiogenin ↔ HSP70	0.45	0.01
Baseline	HM	Angiogenin ↔ ST2	0.40	0.02
Baseline	HM	S100A8/S100A9 ↔ HSP70	0.43	0.01
Baseline	HM	S100A8 ↔ IL33	0.63	<0.001
Baseline	HM	HBDF2 ↔ esRAGE	0.48	0.004
Baseline	M	S100A8/A9 ↔ HMGB1	0.48	0.01
Peak	HM	Angiogenin ↔ S100A8	0.37	0.03
Peak	HM	MBP ↔ HMGB1	-0.42	0.03
Peak	M	S100A8 ↔ AGECML	-0.42	0.034
Peak	M	MBP ↔ esRAGE	0.80	<0.001
Recovery	HM	Angiogenin ↔ S100A8/A9	0.42	0.01
Recovery	HM	HBDF2 ↔ M65M30urine	0.60	0.01
Recovery	M	Angiogenin ↔ M65M30urin	0.52	0.03
Recovery	M	MBP ↔ esRAGE	0.66	<0.01

Table 1: *Correlations of Serum concentrations of antimicrobial peptides with other laboratory parameters in marathoners, half-marathoners at baseline, peak and recovery. Data is given with Correlation Coefficient (R), Values were correlated using Pearson correlation coefficient; HM, half-marathoners; M, marathoners; HMGB1, High mobility group box 1; HSP70, Heat-shock-protein 70 ; esRAGE, endogenous secreted receptor for advanced glycation end-products; MBP, major basic protein; AGECML, Advanced glycation endproducts-carboxymethyllysine, HBDF2, human beta-defensin 2; ST2, suppression of tumorigenicity 2; baseline, 1-2 days before the run; peak, immediately after the run in the finishing area; recovery, after 2-7 days after recovery.*
